# Improved hormonal and oxidative changes by Royal Jelly in the rat model of PCOS: An experimental study

**DOI:** 10.18502/ijrm.v19i6.9373

**Published:** 2021-07-27

**Authors:** Fatemeh Khazaei, Elham Ghanbari, Mozafar Khazaei

**Affiliations:** ^1^Student Research Committee, Kermanshah University of Medical Sciences, Kermanshah, Iran.; ^2^Fertility and Infertility Research Center, Health Technology Institute, Kermanshah University of Medical Sciences, Kermanshah, Iran.

**Keywords:** Royal Jelly, Polycystic ovary syndrome, Ovary, Sex hormone.

## Abstract

**Background:**

Polycystic ovarian syndrome (PCOS) is an endocrine and complex metabolic disorder, associated with anovulation, changes in sex hormone, biochemical factors, and ovarian tissue. Royal Jelly (RJ) has antioxidant and anti-inflammatory properties.

**Objective:**

To examine the therapeutic effect of RJ on PCOS-related hormonal and biochemical changes in a rat model of PCOS.

**Materials and Methods:**

In this experimental study, 42 female Wistar rats (weighing 180–200 gr, aged 10–12 wk) were divided into six groups (n = 7/each): control; PCOS; RJ 100 mg/kg; RJ 200 mg/kg; PCOS + RJ 100 mg/kg; and PCOS + RJ 200 mg/kg. After 21 days, the animals were weighed and dissected. The serums were used for nitric oxide (NO) and ferric-reducing antioxidant power (FRAP) assay and estradiol and progesterone measurements. The ovaries were assessed for histological changes.

**Results:**

PCOS increased estradiol and NO levels, and decreased progesterone and FRAP levels. In PCOS + RJ groups, the progesterone (p = 0.01) and FRAP levels (p ≤ 0.001) increased and the estradiol and NO (p ≤ 0.001) levels decreased significantly. Moreover, the number of mature follicles (p = 0.01) and corpus luteum increased (p ≤ 0.001), and ovarian and uterus weight deceased significantly (p ≤ 0.001).

**Conclusion:**

RJ improved estradiol, progesterone, FRAP, and NO levels, and ovarian structure in the rat model of PCOS.

## 1. Introduction

Polycystic ovarian syndrome (PCOS) is one of the most common endocrine disorders in women of reproductive age with a prevalence of 7–10% (7.1% in Iran). PCOS is associated with infertility, hyperandrogenism, metabolic disorder, deceased life quality, and anovulation. In PCOS women, LH level is increased and their ovaries produce extra normal androgen, which inhibits follicle growth. The pathology of PCOS is complex, and several environmental and genetic factors involved in the hormonal imbalance and hyperandrogenism are a well-established factor in the etiology of this syndrome (1–3).

Oxidative stress (OS) plays an important role in the PCOS and is probably involved in androgen production in the ovary and abnormal follicle formation (4). PCOS induction with estradiol leads to the increased OS in ovarian tissue which in turn reduces the antioxidant capacity of ovary. Therefore, antioxidant supplements may be effective in reducing the symptoms of this disease (5). Inducible nitric oxide (NO) synthase involved in the ovulation process and intraperitoneal NO injection in albino mice lead to a polycystic appearance in the ovary (6). In humans, NO exerts an inhibitory effect on ovarian steroidogenesis (7). Consumption of metformin alone or with clomiphene and letrozole has proven to decrease hyperandrogenic and hyperinsulinemia in PCOS women (8), however, these medications do not threat PCOS completely. Therefore, there is an urgent need for finding and preparing a substitute treatment for PCOS, especially with natural compounds.

Royal Jelly (RJ) is a natural compound with anti-inflammatory, antioxidant, and fertility-stimulation properties (9). It directly stimulates progesterone secretion and has proven to increase sex potential and pregnancy rate in sheep. It can improve hormonal abnormalities, the most important cause of anovulation and defects in uterine growth (10, 11). Our previous study showed that RJ increased the levels of estrogen and progesterone hormones and the number of the mature follicle in immature rats (12). RJ improved blastocyst formation and reduced the rate of apoptosis in cumulus cells (13). Furthermore, it can increase the number of large follicles (4 mm in size) and ovulation in sheep (14). In today's society, PCOS is one of the most common causes of anovulation infertility in women. Thus, using animal models can help find out the causes of this syndrome by conducting PCOS studies.

Given the various properties of RJ, including antioxidant and anti-inflammatory properties, the present study aimed to evaluate the therapeutic effect of RJ on steroid hormone abnormalities and NO changes and the total antioxidant capacity in the ovaries of adult PCOS rat.

## 2. Materials and Methods

### Study design

This experimental study was performed at the Fertility and Infertility Research Center, Kermanshah University of Medical Sciences, Kermanshah, Iran between June and September 2020. Forty-two female Wistar rats (weighing 180–200 gr, aged 10–12 wk) were maintained at 22°C in a 12-hr light/dark cycle with access to standard food and water. RJ was obtained from a local beekeeping association (Tabriz, Iran) and preserved at –4°C until use. It was confirmed by an expert academic member.

### PCOS induction

PCOS was induced by a single intramuscular (groin) injection of estradiol valerate (Aboureihan Pharmacy, Tehran) at 4 mg/kg (dissolved in sesame oil). The time required to develop PCOS was two months after the injection. Vaginal smears were prepared to ensure PCOS. The presence of keratinized cells in the smears is a sign of ovarian follicular cysts. Rats were randomly divided into 6 groups (n = 7/each).

The control and PCOS rats received 0.5 ml of distilled water (RJ solvent) daily by gavage. RJ groups (100 or 200 mg/kg) and PCOS + RJ (100 or 200 mg/kg) groups, were given RJ by gavage daily for 3 wk. At the end of the study, rats were weighed and anesthetized, and blood was taken from the heart and centrifuged for 15 min at 2500 rpm. Serum was then centrifuged and stored at –20°C for the measurement of E2, progesterone, NO, and total antioxidant capacity (FRAP). The ovaries were removed and washed with saline solution, and after separating excess fat, they were immediately fixed in 10% formalin and stained by hematoxylin and eosin method.

### FRAP assay

The serum total antioxidant capacity was determined by the FRAP method. This method is based on the capacity of the sample antioxidants (serum) in the reduction of Fe${}^{+3}$ to Fe${}^{+2}$ ions in the presence of tri-pyridyl triazine. The absorption changes of samples were measured by a spectrophotometer (Pharmacia, Novaspec II, Biochrom, England) at 593 nm (15).

### NO assay

Serum NO was assessed using the Griess colorimetric method. Because of the NO instability, its direct measurement is difficult, the nitrite and nitrate levels of the various liquids are considered as NO indicators. Briefly, 400 μL of serum samples were deproteinized by adding 6 mg of zinc sulfate and centrifuged for 15 min at 12000.

Then 100 µl of the supernatant, 100 µl of the vanadium chloride solution, and Griess solution containing sulfanilamide and N-(1-naphthyl) ethylene diamine di-hydrochloride were added and incubated in 37°C for 30 min. The samples were read at 450 and 630 nm, the absorbance were compared to the standard absorbance (0–200 μM sodium nitrate), and the concentration of the samples was calculated (16).

### Histological examination

The ovaries were fixed and prepared to obtained serial 5-µm sections and stained by the hematoxylin–eosin method. Each section was photographed using a light microscope equipped with a Motic photographic camera and software (Moticam 2000, Motic, Spain). Two researchers separately determined the average tissue changes, including the types and numbers of follicles and the corpus luteum in the ovaries (12, 17).

### Ethical considerations

All animal experiments were approved by the Institutional Animal Ethics Committee of Kermanshah University of Medical Sciences, Kermanshah, Iran (Code IR.KUMS.REC.1399.413) and conformed to the Guide for the Use and Care of Laboratory Animals.

### Statistical analysis

Normal distribution of data was determined by the Kolmogorov–Smirnov method and data were analyzed by the Kruskal–Wallis test and one-way ANOVA, and Tukey's post hoc test using SPSS software (version 18, Inc. Chicago, IL, USA). Data was presented as mean ± SE and P-values < 0.05 were considered significant.

## 3. Results

**Figure 1:**
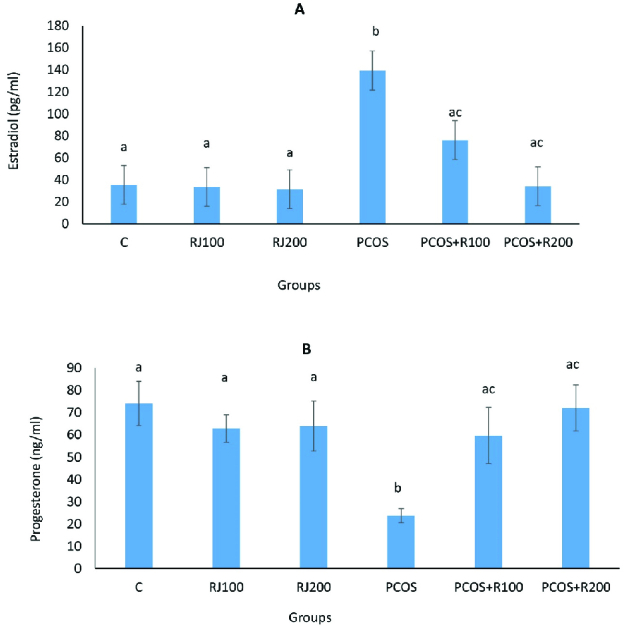
Comparison of the effect of RJ on the serum levels of E2 (A) and progesterone (B) in the control and experimental groups. Data presented as Mean ± SE. Groups with different superscript letters are statistically significant (one-way analysis of variance, Tukey's post hoc, p < 001).

**Figure 2:**
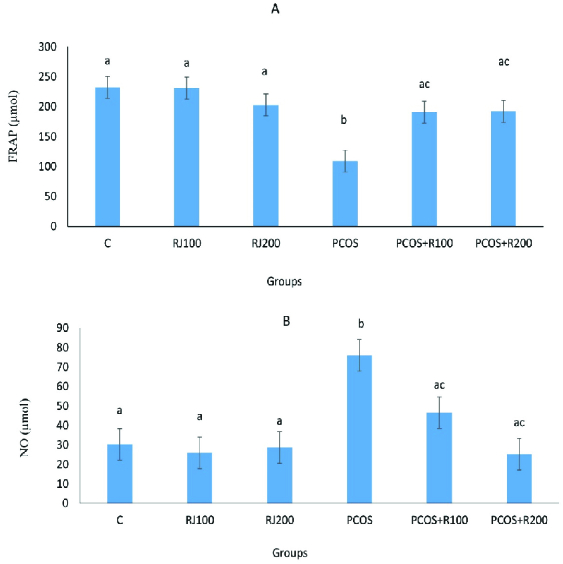
Comparison of the effect of RJ on FRAP (A) and NO levels (B) in the control and experimental groups. Data presented as Mean ± SE. Groups with different superscript letters are statistically significant (one-way analysis of variance, Tukey's post hoc, p < 0.001).

E2 increased significantly (p ≤ 0.001) in the PCOS groups, with a dose-dependent decrease in RJ + PCOS groups, and reached the control level at RJ 200 mg/kg. Normal rats treated with RJ (100 and 200 mg/kg) did not show a significant change (Figure 1A). P-values were significantly decreased (p = 0.01) in the PCOS group but significantly increased in the RJ + PCOS groups in a dose-dependent manner. Normal rats treated with RJ did not show a significant difference with the control group (Figure 1B). Serum levels of FRAP were significantly decreased (p ≤ 0.001) in the PCOS group while it was significantly increased in the RJ+PCOS groups. Normal rats treated with RJ had no significant difference with the control group (Figure 2A). Serum levels of NO were also significantly increased in the PCOS group (p ≤ 0.001) and significantly decreased in the RJ + PCOS groups (Figure 2B).

**Figure 3:**
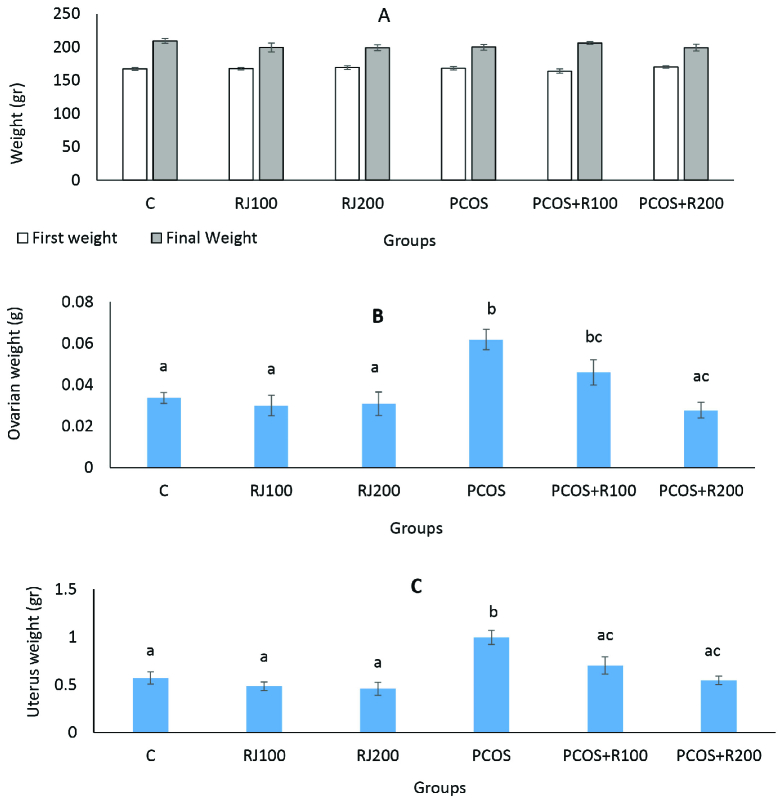
Comparison of the effect of RJ on body weight (A) (p = 0.48), ovarian weight (B), and uterine weight (C) (p ≤ 0.001) in the control and experimental groups. Data presented as Mean ± SE. Groups with different superscript letters are statistically significant (one-way analysis of variance, Tukey's post hoc, p < 0.001).

At the end of the experiment, the mean body weight of rats were not significantly different (p = 0.48) (Figure 3A). However, the ovarian weight was significantly increased (p ≤ 0.001) in the PCOS group, and a dose-dependent decrease in RJ + PCOS groups was seen. Normal rats treated with RJ did not show a significant difference with the control group (Figure 3B). Uterine weight was significantly increased in the PCOS group (p ≤ 0.001) and a dose-dependent decrease was seen in the RJ + PCOS groups. Normal rats treated with RJ did not show significant difference with the control group (Figure 3C).

**Figure 4:**
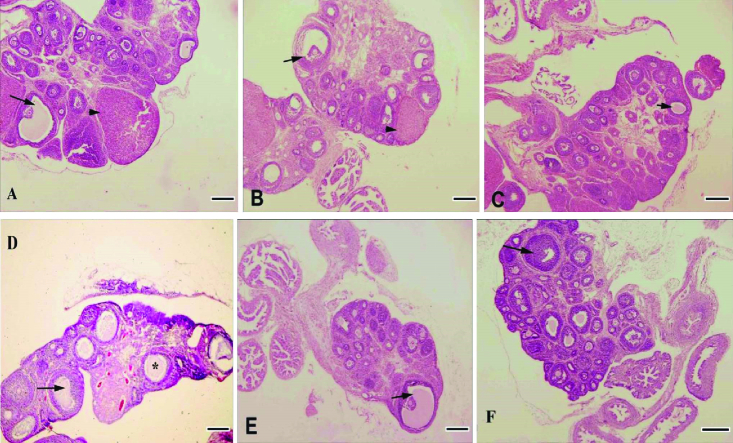
Light micrograph of histopathological sections of the ovary (H&E; magnification ×4). (A) Control groups, (B) RJ (100 mg/kg), and (C) RJ (200 mg/kg) groups showed normal ovary histological structure and (D) PCOS, (E) PCOS + RJ 100 mg/kg, and (F) PCOS + RJ 200 mg/kg groups showed normal structure without signs of pathology in the ovarian. Arrow: Graafian follicle, Arrowhead: Number of corpora lutea, *Cystic follicles. Scale Bar: 200 µm.

Control and RJ groups (100 and 200 mg/kg) showed a normal ovarian histological structure (Figure A, B, C), but the number of mature follicles and corpus luteum were decreased in the PCOS groups significantly (p < 0.01) and the number of large cystic follicles increased (Figure 4D). Treatment of PCOS rats with RJ resulted in an increase in the number of mature follicles even more than in the control group and an increase in the number of corpus luteum (Table I). RJ increased the number of adult follicles in normal rats (Figures 4E, F). PCOS affected the ovarian structure by increasing large-sized cysts instead of secondary and tertiary follicles and decreasing the number of corpus luteum (Figure 4D). The number of secondary, tertiary, and graafian follicles were decreased in PCOS rats. However, RJ-treated rats showed a reduction in the number of cystic follicles and normal structures without signs of pathology in the ovaries (Figure 4E, 4F).

**Table 1:** Comparison of the effect of RJ on the number of corpus luteum and mature follicles in the control and experimental groups


**Groups**	**Number of corpora lutea**	**Number of graafian follicle**
**C**	4.5 ± 0.43${}^{a}$	2 ± 0.45${}^{a}$
**RJ100**	3.67 ± 0.67${}^{a}$	3.67 ± 0.67${}^{a}$
**RJ200**	4.17 ± 0.48${}^{a}$	2.33 ± 0.61${}^{a}$
**PCOS**	0 ± 0${}^{b}$	0.67 ± 0.33${}^{b}$
**PCOS + RJ100**	3 ± 1.03${}^{a}$	3.33 ± 0.71${}^{a}$
**PCOS + RJ200**	3.5 ± 0.76${}^{a}$	3 ± 0.68${}^{a}$
Data presented as Mean ± SE. Groups (columns) with different superscript letters (${}^{a,b}$) are statistically significant (one-way analysis of variance, Tukey's post hoc, p < 0.05). RJ: Royal Jelly, PCOS: Polycystic ovarian syndrome		

## 4. Discussion

In the present study, treatment of PCOS rats with RJ improved serum antioxidant status, progesterone, E2, and NO levels and increased the number of mature follicles and corpus luteum. In most of the studied factors, a dose of RJ 200 mg/kg showed better effects, and the variables were near to the control group (12, 18). RJ also improved ovarian structure in PCOS rats in terms of follicles type and corpus luteum formation. To the best of our knowledge, this is the first report on the improving effect of RJ on PCOS complications, which is in line with our previous study in which RJ increased ovulation in immature rat (12). PCOS is one of the most important conditions in which follicular atresia and cessation of growth are observed (4). One of the important diagnostic criteria for PCOS is an alteration of sex hormone levels, including increased estrogen and decreased progesterone (2), causing these endocrine abnormalities to underlie ovulation and infertility. In the present study, similar to other studies, E2 levels increased and progesterone levels decreased, and treatment with different doses of RJ improved these changes (3, 19).

In biological systems, the imbalance between reactive oxygen species and antioxidant defense systems, including glutathione peroxidase, superoxide dismutase, and catalase enzymes, leads to OS. Consequently, assays such as FRAP are used to evaluate total antioxidant capacity. FRAP is one of the most common parameters to measure this process (20). It was showed that OS helps to develop PCOS in women and that reducing total antioxidant capacity increases the oxidation products in PCOS. They believe that these patients should take antioxidant supplements such as vitamins C and E to achieve a proper antioxidant defense system and reduce the harmful effects of OS (21). In the present study, RJ increased total antioxidant capacity in PCOS rats, which can be attributed to its antioxidant properties.

Studies have shown that increased concentrations of NO or NO-releasing agents can prevent the production of steroids in luteal and granulosa cells in humans and laboratory animals (6, 22). This can increase the levels of ovarian androgens and consequently result in the cystic ovary. On the other hand, some NO-mediated cytotoxicity has been demonstrated in the ovaries (23, 24). In the present study, NO levels were reduced in RJ-treated PCOS groups. Therefore, RJ may decrease NO levels in PCOS rats, which may contribute to the decrease in the level of androgens secreted from the ovaries and thus to the elimination of cysts in the ovaries.

It was also reported that the antioxidant compounds of the RJ by affecting the synthesis of intrinsic antioxidants can be effective in the production and protection of intracellular antioxidants and ultimately eliminate free radicals (25). In the present study, treatment with RJ for 21 days in PCOS rats improved hormonal abnormalities and increased total antioxidant capacity. Antioxidant compounds can reduce OS in ovarian tissue, and significantly reduce the number of cystic follicles (26). In our previous study, RJ increased the number of mature and antral follicles and the appearance of the corpus luteum in immature rat ovaries (12). In line with that report, in the present study, treatment with RJ increased the number of follicles and corpus luteum formation and decreased the number of cystic follicles in PCOS.

Studies have shown that in PCOS rats, uterine and ovarian weight increase due to hormonal imbalance (18, 27), which was consistent with the present study. Furthermore, the inflammatory effects of NO and its role in the production of prostaglandins in the uterus have been documented. On the other hand, E2 and progesterone, through systems such as NO, increase angiogenesis in the endometrium and raise secretory gland activity (28). In addition, in the present study, increased uterine weight was observed in the PCOS group, which may be due to inflammation caused by high concentrations of androgenic hormones. During treatment with RJ, the weight of the uterus decreases, this may be due to reduced inflammation due to decreased androgen levels or RJ antioxidant properties.

## 5. Conclusion

RJ improved hormonal status and increased the number of Graafian follicles in PCOS rats with its antioxidant effects. Therefore, it can be considered as an effective natural product for improving the symptoms of PCOS.

##  Conflict of Interest

The authors declare that there is no conflict of interest in this study.
